# Investigation of strain and doping on the electronic properties of single layers of C_6_N_6_ and C_6_N_8_: a first principles study†

**DOI:** 10.1039/d0ra04463f

**Published:** 2020-07-24

**Authors:** Asadollah Bafekry, Chuong V. Nguyen, Abbas Goudarzi, Mitra Ghergherehchi, Mohsen Shafieirad

**Affiliations:** Department of Physics, University of Guilan 41335-1914 Rasht Iran Bafekry.asad@gmail.com; Department of Physics, University of Antwerp Groenenborgerlaan 171 B-2020 Antwerp Belgium; Institute of Research and Development, Duy Tan University Da Nang 550000 Vietnam; Department of Physics, University of North Texas Denton Texas USA; College of Electronic and Electrical Engineering, Sungkyunkwan University Suwon Korea mitragh@skku.edu; Department of Electrical and Computer Engineering, University of Kashan Kashan Iran

## Abstract

In this work, by performing first-principles calculations, we explore the effects of various atom impurities on the electronic and magnetic properties of single layers of C_6_N_6_ and C_6_N_8_. Our results indicate that atom doping may significantly modify the electronic properties. Surprisingly, doping Cr into a holey site of C_6_N_6_ monolayer was found to exhibit a narrow band gap of 125 meV upon compression strain, considering the spin–orbit coupling effect. Also, a C atom doped in C_6_N_8_ monolayer shows semi-metal nature under compression strains larger than −2%. Our results propose that Mg or Ca doped into strained C_6_N_6_ may exhibit small band gaps in the range of 10–30 meV. In addition, a magnetic-to-nonmagnetic phase transition can occur under large tensile strains in the Ca doped C_6_N_8_ monolayer. Our results highlight the electronic properties and magnetism of C_6_N_6_ and C_6_N_8_ monolayers. Our results show that the electronic properties can be effectively modified by atom doping and mechanical strain, thereby offering new possibilities to tailor the electronic and magnetic properties of C_6_N_6_ and C_6_N_8_ carbon nitride monolayers.

## Abstract 2

Using first-principles calculations, we explore the effects of atom doping and strain on the structural, electronic, and magnetic properties of C_6_N_6_ and C_6_N_8_ monolayers.

## Graphical abstract

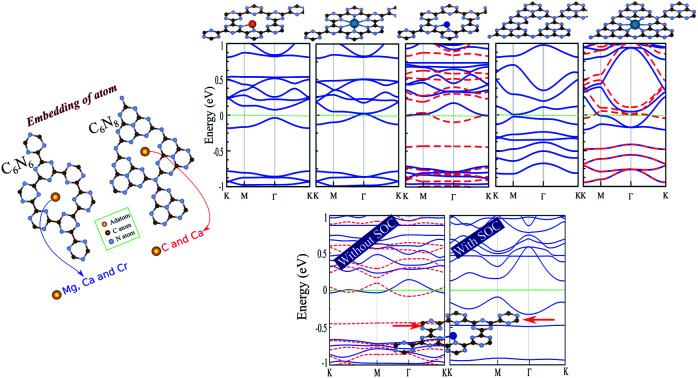

## 1 Introduction

Recently, two-dimensional carbon nitride (2D-CN) nanomaterials have attracted remarkable attention, not only because of astonishing experimental advances concerning their synthesis but also due to their exciting physics^1–5^ and promising prospects for various advanced applications, like nanoelectronics, energy storage and catalysis.^6–16^ Two-dimensional carbon nitride (2D-CN) nanomaterials belong to a group of allotropes with a common chemical formula of C_*n*_N_*m*_, where *n* and *m* represent the number of C and N atoms in the unit cell, respectively, which can be stabilized as monolayers by taking advantage of the multifarious chemistry of C and N atoms. Depending on the composition of C and N atoms in the atomic lattice, these allotropes can show diverse electronic properties, ranging from semiconducting to half-metallic. 2D-CN monolayers exhibit considerable structural, electronic and magnetic properties^17–21^ with diverse electronic properties, originating from their unique and different atomic lattices, made from strong covalent bonds.^22–25^

The successful synthesis and fabrication of two-dimensional carbon nitride (2D-CN) nanomaterials, including C_2_N, C_3_N, C_6_N_8_ and C_6_N_6_ through bottom-up procedures, has motivated researchers to consider possible approaches for tuning the band gap and electronic response of these attractive nanosheets.^22,26–28^ Some 2D-CN nanosheets have been experimentally realized using different approaches.^1,3,29–33^ C_6_N_6_ is a 2D-CN allotrope in which two hexagonal rings are separated by a C–C bond,^1^ and it has already been theoretically investigated.^34–36^ Recent theoretical work showed that C_6_N_6_ exhibits a topologically nontrivial band gap, which can be modulated to a topological insulator by doping.^1^ To search for a suitable spintronic material such as a diluted magnetic semiconductor, first-principles calculations of transition metal (TM) atoms doped in semiconducting 2D-CN sheets have been performed^2,37,38^ using density functional theory (DFT) calculations.^39,40^ The semiconducting nature and band alignment in this nanosheet are suitable for hydrogen generation *via* photo-catalytic water splitting.^41–44^

First-principles calculations indicated that topologically nontrivial electronic states near the Fermi level can be characterized by a ruby model.^45^ Recently, carbon nitride materials with stoichiometry of C_6_N_8_ have been synthesized experimentally.^12,26,46,47^ C_6_N_8_ are the prevailing building blocks that are joined together directly or by sp^2^-hybridized nitrogen/carbon atoms.^12^ The electronic and magnetic properties have subsequently been studied,^48,49^ and theoretical studies demonstrate that C_6_N_8_ is not a magnetic material; however, after replacing a nitrogen atom with a carbon atom in the unit cell of C_6_N_8_, it displays intrinsic half-metallicity.^3^ Hydrogenation can also induce magnetism in carbon-based nonmagnetic materials.^50,51^ It was predicted theoretically that hydrogen doping on the irradiation-induced carbon vacancy with hydrogen adsorption could induce a stronger magnetic moment than the bare vacancy.^52^ Moreover, first-principles calculations also show that semihydrogenated graphene sheets are ferromagnetic semiconductors with a small indirect gap.^53^ On the other hand, it is well-known that the application of mechanical strain can be an effective way to adjust the electronic structure of 2DM,^54^ and in previous studies strain has been proven as a common way to regulate properties. In order to enhance the application of a material in electronic and optoelectronic devices, it can be very useful to regulate the electronic properties. Atom impurities can tailor the electronic properties and can be used for a wide range of applications. Despite the fact that many 2D nanosheets have been synthesized recently, their tunable electronic properties have rarely been reported.^55–90,108–110^

In this paper, using first-principles calculations, we systematically investigate the effect of different atom impurities in the holey sites of C_6_N_6_ and C_6_N_8_ monolayers. Our results confirm that the electronic properties can be modified by doping with Cr atoms. In addition, by applying strain, we found that the magnetic moment can be modulated and a transition from semiconductor to metal can be achieved. Our results reveal that the band gap and magnetism can be modified or induced by various atom impurities, thereby offering effective possibilities to tune the electronic and magnetic properties of C_6_N_6_ and C_6_N_8_ monolayers.

## 2 Methods

We report the results of our spin-polarized DFT calculations for the electronic structure as implemented in the OpenMX 3.8 package.^91^ Wave functions were expanded by the linear combination of multiple pseudoatomic orbitals (LCPAOs) created using a confinement scheme.^92,93^ The generalized gradient approximation method proposed by Perdew–Burke–Ernzerhof (GGA-PBE)^94^ was applied to deal with the exchange-correlation functionals, along with the norm-conserving pseudopotentials.^95^ A quasi-Newton algorithm was employed to find the energy-minimized structures using the OpenMX package. The convergence criteria for the structural optimization were set as a force of less than 1 meV Å^−1^ acting on each atom. After the completion of this step, we used a cutoff energy of 300 so that the total energies converge to below 1.0 meV per atom. The Brillouin zone was sampled by a *k*-point grid of 23 × 23 × 1 for the primitive unit cell and scaled according to the size of the supercells, on the basis of the Monkhorst–Pack scheme.^96^ The monolayers are modelled as a periodic slab with a large vacuum region (20 Å) in order to prevent interactions between adjacent layers. To describe the vdW interactions we use the empirical correction method described by Grimme (DFT-D2).^97^ The charge transfer was calculated using Mulliken charge analysis.^98^ Scanning tunneling microscopy (STM) images were simulated using the Tersoff–Hamann theory.^99^ The STM simulated images were plotted using WSxM software.^100^

## 3 Results

### 3.1 Pristine C_6_N_6_ and C_6_N_8_ monolayers

**Fig. 1:**
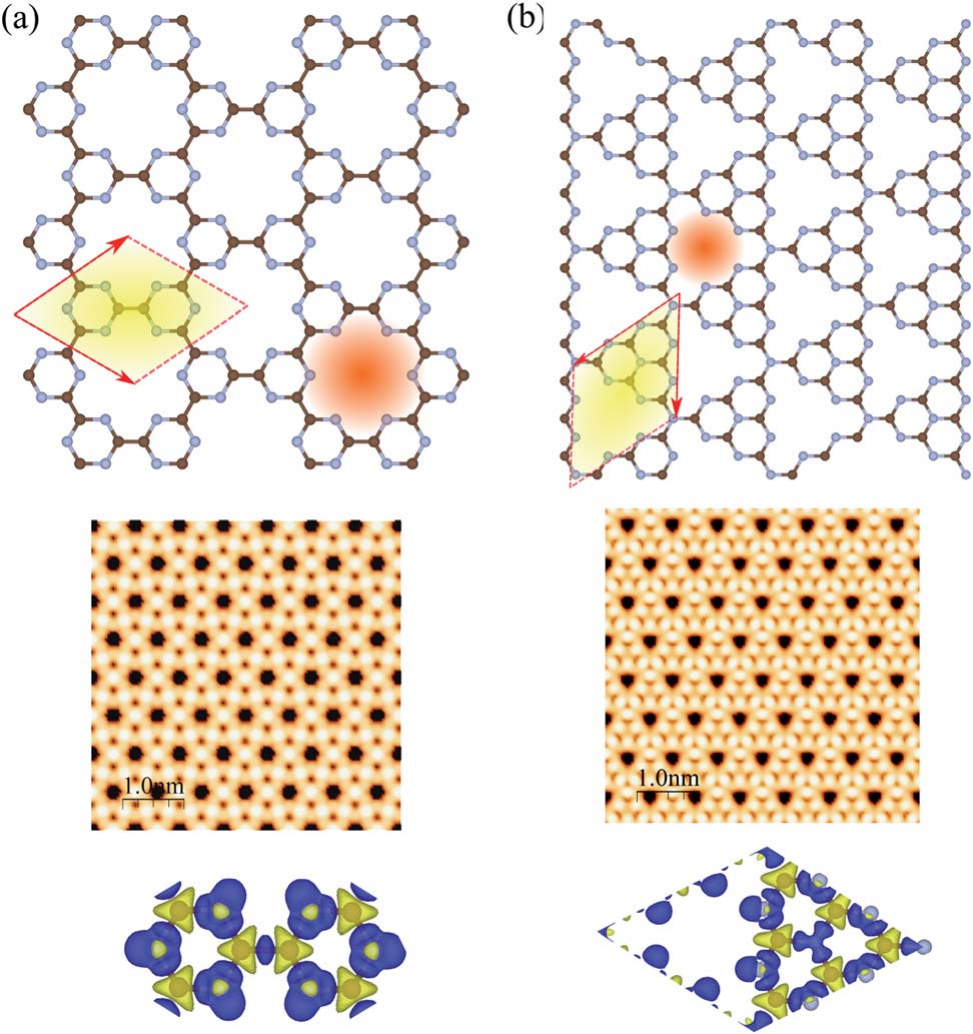
Atomic structures (top), simulated STM images (middle) and charge density differences (bottom) of (a) C_6_N_6_ and (b) C_6_N_8_ monolayers. The primitive unit cells are indicated by red parallelograms. The brown and blue atoms represent C and N atoms, respectively. The blue and yellow regions represent the charge accumulation and depletion regimes, respectively.

**Fig. 2:**
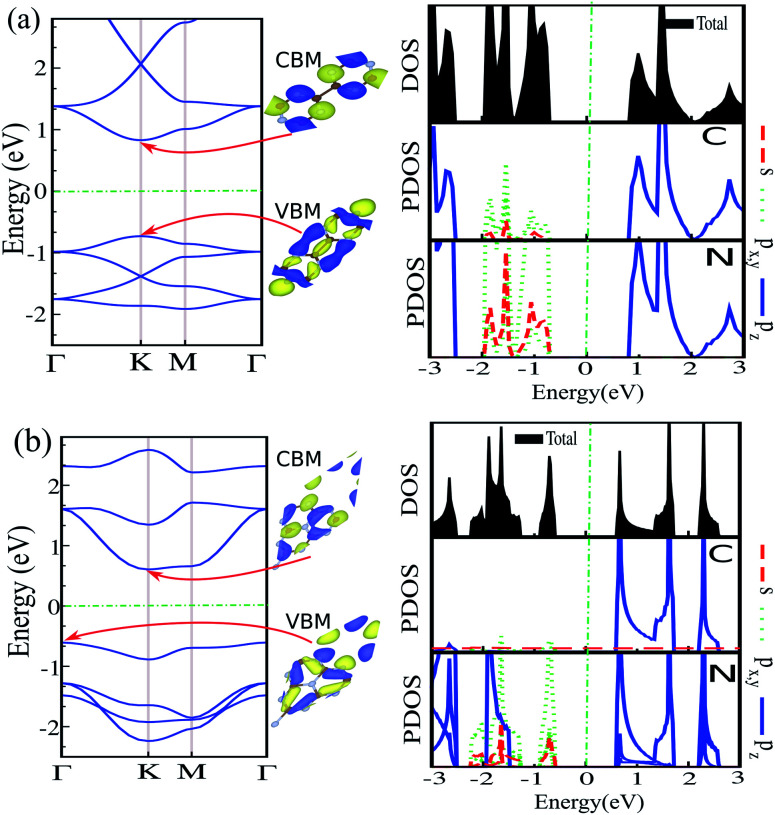
Electronic band structure (left), density of states and partial density of states (right) of (a) C_6_N_6_ and (b) C_6_N_8_ monolayers. The insets represent the charge densities of the VBM and CBM. Zero energy is set at the Fermi level.

The atomic structures, simulated STM images, and charge density differences of C_6_N_6_ and C_6_N_8_ monolayers are shown in Fig. 1(a) and (b), respectively. The optimized lattice constant of C_6_N_6_ is calculated to be 7.11 Å and the corresponding C–C and C–N bond lengths are found to be 1.503 Å and 1.343 Å, respectively. The nanopore diameter is found to be 5.447 Å, which is equivalent to the distance from one N atom to the opposite N atom across the pore. These results are consistent with previous reports.^36,101–103^ The charge density difference, in which the blue and yellow colors show the charge accumulation and depletion, respectively, reveals that the positively charged C atoms are surrounded by negatively charged N atoms. Moreover, the simulated STM image indicates that the atoms around the C atom sites show bright spots. The electronic band structure, density of states (DOS) and partial DOS (PDOS) of C_6_N_6_ and C_6_N_8_ monolayers are shown in Fig. 2(a) and (b), respectively. Apparently, the C_6_N_6_ monolayer is a semiconductor with a direct band gap of 1.5 eV located at the K-point, which is also supported by a previous report.^36^ From the PDOS, we found Fermi-level sharp peaks +2 eV below the Fermi level, suggesting that there are localized wave functions in C_6_N_6_ and indicating that sharp double peaks (flat shallow bands) are formed from the N s and p_*x*,*y*_ orbitals. In the VBM, the N atoms are in the sp^2^ configuration and their lone pairs are further exhibited in the rising band structure from the Γ to the K point near the Fermi-level. The lone pairs of N s and p_*x*,*y*_ lie in in-plane orbitals, suggesting the donor nature of the nanopore, although the N/C p_*z*_ antibonding delocalized orbitals form the CBM above the Fermi-level, showing the acceptor nature of C_6_N_6_. The filling of all the in-plane bonding orbitals implies high stability of the C_6_N_6_ structure.

The optimized lattice constant of monolayer C_6_N_8_ is found to be 7.14 Å, with two inequivalent C–N bonds (1.414 and 1.455 Å). In addition, the ring is not a regular hexagon since the C–N–C angle is equal to 120°, while the N–C–N angles are 118° and 122°, respectively. It is clear that every six C–N hexagon rings enclose a nanopore, while the edges are surrounded by six N atoms, with a nanopore diameter of 4.773 Å (see Fig. 2(b)), which is in agreement with previous reports.^39,101,104,105^ The electronic structure of C_6_N_8_ exhibits an indirect semiconductor with a 1.22 eV band gap, which is in agreement with the previous result.^106^ In addition, from the PDOS of C_6_N_8_ it can be clearly seen that the VBM is determined by the N s and p_*x*,*y*_ orbitals; however, the CBM originates from the N/C p_*z*_ orbitals.

### 3.2 Doping of atom into holey site

Here, we discuss the effect of atom doping on the structural and electronic properties of C_6_N_6_ and C_6_N_8_ monolayers. A 2 × 2 × 1 supercell of C_6_N_6_ and C_6_N_8_ contains 49 and 57 atoms, respectively. A schematic view of the doped atom in the holey site of the C_6_N_6_ and C_6_N_8_ monolayers is shown in the ESI, Fig. S1.† For the different atoms considered in our present study, the stable binding sites are uniformly at the nanopore. Through the rest of the study, the atom-doped C_6_N_6_ structure is labeled as atom@C_6_N_6_. For instance, Mg doped C_6_N_6_ is labeled Mg@C_6_N_6_.

**Fig. 3:**
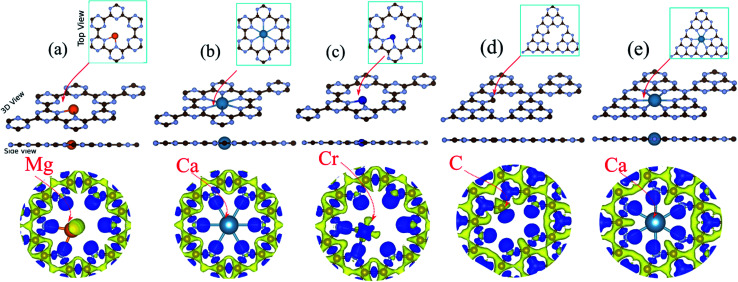
Optimized structures of (a) Mg, (b) Ca, and (c) Cr atom doped C_6_N_6_. (d and e) Optimized structures of C and Ca doped C_6_N_8_. Charge density differences are shown in the bottom panel. Blue and yellow regions represent charge accumulation and depletion, respectively.

The optimized structures of Mg@, Ca@ and Cr@C_6_N_6_ are shown in Fig. 3(a–c), respectively. The Mg and Ca atoms interact through sp^2^-hybridization and form two and six σ bonds with the neighboring N atoms, respectively. Our results show that the bond lengths of the Mg and Ca atoms with the nearest N atoms are 2.62 and 2.37 Å, respectively (see Table 1). The Cr atom binds to the two nearest N atoms facing the nanopore of the C_6_N_6_ monolayer with a bond length of 1.94 Å. Notably, upon atom doping, the planar structure of monolayer C_6_N_6_ is preserved. The charge density differences are presented in Fig. 3, where the bond formation, charge accumulation, and depletion regions can clearly be seen. The blue and yellow regions represent charge accumulation and depletion, respectively. For the stability of the structures, we calculate the binding energy (*E*_b_) between the doped atoms and the C_6_N_6_/C_6_N_8_ sheets by the following formula:

1 *E*_b_ = *E*_tot_ − *E*_sheet_ − *E*_atom_

Here, *E*_tot_ is the total energy of the doped C_6_N_6_/C_6_N_8_ sheet, and *E*_sheet_ and *E*_atom_ are the energies of the pristine C_6_N_6_/C_6_N_8_ sheet and isolated spin-polarized atom, respectively. According to this definition, a more negative value of *E*_b_ indicates that the system has stronger binding between the atom and the C_6_N_6_/C_6_N_8_ sheet. Our results show that the binding energies for C, Mg, Ca and Cr atom doping in C_6_N_6_ (C_6_N_8_) sheets are −3.46 eV (−3.38), −3.70 eV (−3.43), −3.95 eV (−3.87) and −4.65 eV (−3.65).

**Table 1:** Structural, electronic and magnetic parameters of atom doped C_6_N_6_ and C_6_N_8_ monolayers. Bond length between doped atom and its nearest atom (*d*_AN_); bond length between N–C atoms (*d*_NC_); bond length between C–C atoms (*d*_CC_); charge transfer (Δ*Q*); magnetic moment per supercell *M*_tot_ (*μ*_B_). Electronic state (ES), specified as metal (M), half-metal (HM), ferromagnetic metal (FM), semiconductor (SC). The values outside (inside) parentheses are for C_6_N_6_ (C_6_N_8_) monolayers

Atom	*d*_AN_ (Å)	*d*_NC_ (Å)	*d*_CC_ (Å)	Δ*Q* (e)	*M*_tot_ (*μ*_B_)	ES
C	1.46 (1.39)	1.40 (1.44)	1.42 (1.42)	0.99 (1.38)	0 (0)	SC (SC)
Mg	2.16 (2.30)	1.39 (1.41)	1.44 (1.44)	0.99 (1.38)	0 (0)	SC (M)
Ca	2.66 (2.37)	1.38 (1.38)	1.43 (1.44)	1.17 (1.32)	0 (1)	M (FM)
Cr	1.94 (2.16)	1.41 (1.40)	1.42 (1.42)	0.56 (0.63)	2.4 (2.1)	FM (HM)

**Fig. 4:**
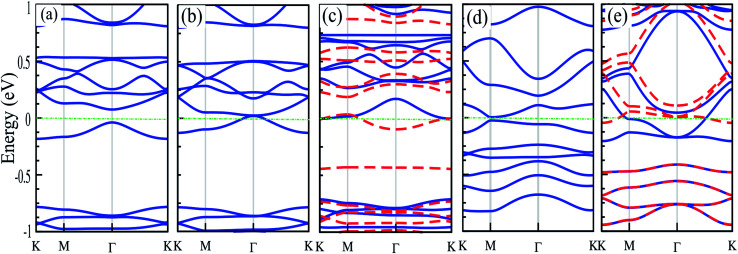
Electronic structures of (a) Mg@C_6_N_6_, (b) Ca@C_6_N_6_, (c) Cr@C_6_N_6_, (d) C@C_6_N_8_ and (e) Ca@C_6_N_8_. The energy bands of ↑ and ↓ spin channels are indicated by blue lines and red dashed lines, respectively. Zero energy is set at the Fermi-level.

**Fig. 5:**
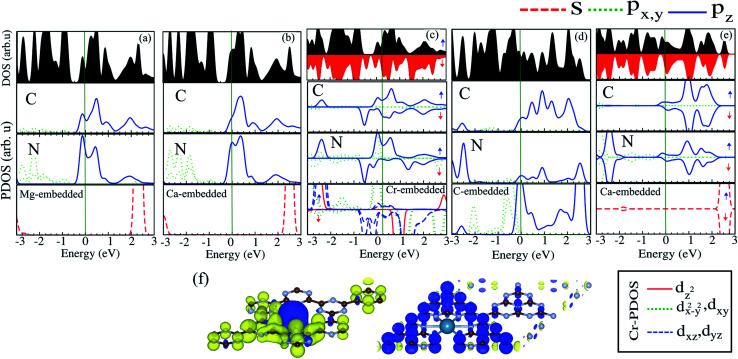
DOS and PDOS of (a) Mg@C_6_N_6_, (b) Ca@C_6_N_6_, (c) Cr@C_6_N_6_, (d) C@C_6_N_8_ and (e) Ca@C_6_N_8_. (f) Spin density difference. The blue and yellow regions represent the ↑ and ↓ spin states, respectively.

The electronic structures of Mg@, Ca@ and Cr@C_6_N_6_ are shown in Fig. 4(a–c), where the solid blue and dashed red lines represent ↑ and ↓ spin channels, respectively. Our results show that in comparison with pristine C_6_N_6_, the band structures are modified and give rise to localized mid-gap states arising from the atomic doping. Note that the doping of Mg and Cr atoms gives rise to some localized states in the band structure, which modify the electronic properties. Our results show that Mg@C_6_N_6_ exhibits direct band gap semiconducting behavior, with a small band gap of 100 meV where the VBM and CBM are located at the Γ point. Interestingly, we can see that the Ca@C_6_N_6_ monolayer exhibits metallic character. In contrast, Cr@C_6_N_6_ is a ferromagnetic metal with a net magnetic moment of 2.4 *μ*_B_. Note that the energy bands of the ↑ and ↓ spin channels near the Fermi-level cross together near to the Dirac-point. On the other hand, monolayer C@C_6_N_8_ becomes a direct band gap semiconductor with a narrow band gap of 25 meV, with the VBM and CBM located at the M point. With Ca atom doping in C_6_N_8_, we see a spin-polarized ferromagnetic metal with impurity levels crossing the Fermi-level and inducing a net magnetic moment of 1 *μ*_B_ per formula unit. The atomic contributions to the electronic states can be further seen by the PDOS, which are presented in Fig. 5. It is clear that the Mg and Ca states in C_6_N_6_ mostly contribute to the deep valence and conduction states. On the other hand, Cr in C_6_N_6_ and C in C_6_N_8_ also contribute to the states around the Fermi-level. The optimized structures and electronic structures of C@C_6_N_6_, Mg@C_6_N_8_ and Cr@C_6_N_8_ are shown in Fig. S2(a–c),† respectively. We found that the C atoms interact through sp^2^-hybridization and form two σ bonds with the neighboring N atoms of C_6_N_6_. The C@C_6_N_6_ structure shows semiconductor behavior with an indirect band gap of 1 eV, where the VBM and CBM are located at the K and M points, respectively (see Fig. S2(a)†). Our results show that the Cr atom bonds to the N host atoms of C_6_N_8_ and forms six σ bonds, while the neighboring N atoms of C_6_N_8_ form four σ bonds with the Mg atom (see Fig. S2(a) and (b)†). From the electronic band structure, we can see that Mg@C_6_N_8_ turns into a metal, whereas the Cr@C_6_N_8_ structure becomes a half-metal and a magnetic moment of about 2.1 *μ*_B_ is induced.

## 4 Effect of strain

**Fig. 6:**
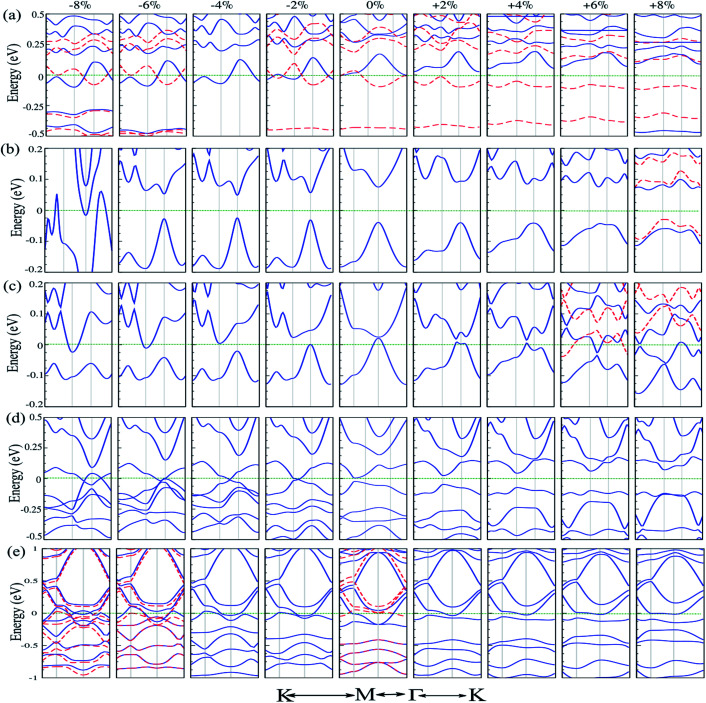
Electronic band structure of (a) Cr@C_6_N_6_, (b) Mg@C_6_N_6_, (c) Ca@C_6_N_6_, (d) C@C_6_N_8_ and (e) Ca@C_6_N_8_ as a function of strain. The energy bands of ↑ and ↓ spin channels are indicated by blue lines and red dashed lines, respectively. Zero energy is set at the Fermi-level.

Strain engineering is a robust method to tune the electronic properties and topological nature. In the following, we investigate the effects of uniaxial tensile and compressive strains on the electronic properties of atom doped C_6_N_6_ and C_6_N_8_ monolayers. The strain is defined as:^107^*ε* = (*a* ± *a*_0_)/*a*_0_ × 100, where *a* and *a*_0_ are the strained and non-strained lattice constants, respectively, and the positive (negative) sign denotes tensile (compressive) strain. The band structures of Cr@, Mg@ and Ca@C_6_N_6_, and C@ and Ca@C_6_N_8_ as a function of the applied strain are shown in Fig. 6(a–e). Cr@C_6_N_6_ displays dilute magnetic semiconductor behavior under +2% tensile strain and it is preserved up to +8% strain. In addition, the magnetic moment increases from 2.8 (at −2%) to 3 *μ*_B_ (at −8%) with increasing compressive strain, while it is almost constant under tensile strain. Interestingly, when the compression strain reaches −2%, Cr@C_6_N_6_ exhibits spin-polarized semi-metal characteristics.

Our results show that Mg@C_6_N_6_ in the range of −2% to −6% exhibits a decreasing band gap from 90 meV to 70 meV, respectively, while in the strain-free case the band gap is 110 meV. This situation differs under tensile strain, such that the band gap changes in the range of 130 meV to 115 meV. Interestingly, at a tensile strain larger than +8%, the band structure of Mg@C_6_N_6_ in both ↑ and ↓ spin channels becomes spin-polarized, leading to the transition from nonmagnetic to magnetic states (see Fig. 6(b)). Ca@C_6_N_6_ is naturally a metal; surprisingly, with strains of −2% and −4% the band gaps reach 25 meV and 23 meV, respectively (see Fig. 6(c)), whereas under strain larger than −6% it transforms into a metal and we see a nontrivial band gap of 30 meV in the vicinity of the Fermi-energy. On the other hand, for monolayer Ca@C_6_N_6_ under +6 and +8% strain, the VBM (CBM) of the ↓ (↑) spin channel continuously shifts upward (downward) to the Fermi level, resulting in ferromagnetic metallic behavior with magnetic moments of 0.8 *μ*_B_ (+6%) and 1.8 *μ*_B_ (+8%). The electronic band structure of C@C_6_N_8_ under strain is shown in Fig. 6(d). C@C_6_N_8_ is initially a direct semiconductor with a narrow band gap of 30 meV; however, under 0% ≤ tensile strain ≤ +8%, the band gap increases monotonically from 70 meV (at +2%) to 200 meV (at +8%), while the VBM and CBM of the direct band gap are located at the M point. Our results show that the semiconducting characteristics are preserved in the range of +2% to +8%. Interestingly, C@C_6_N_8_ under compression strains of −2, −4 and −8% exhibits semi-metal behavior. When it reaches −6%, it transforms to a metal due to the CBM decrease and VBM increase to the Fermi-level. The electronic band structure of Ca@C_6_N_8_ under uniaxial strain in the ↑ and ↓ spin channels is shown in Fig. 6(e). We can see that variations in the magnetic moment occur when it is subjected to tensile and compressive strain. Ca@C_6_N_8_ is initially a ferromagnetic metal with a magnetic moment of 1 *μ*_B_ in the ground state. Interestingly, when a strain larger than +2% to +8% is applied, the ferromagnetic metal state is lost and both the ↑ and ↓ spin channels become non-spin-polarized, leading to a transition from a ferromagnetic metal to a nonmagnetic-metal state. Note that by increasing strain up to +2%, its magnetic moment vanishes. Also, for strains of 2–4%, Ca@C_6_N_8_ transforms into a nonmagnetic metal, while for larger strains (>−6%) the structure transforms into a ferromagnetic metal with magnetic moments of 0.47 *μ*_B_ (−6%) and 0.53 *μ*_B_ (−8%).

**Fig. 7:**
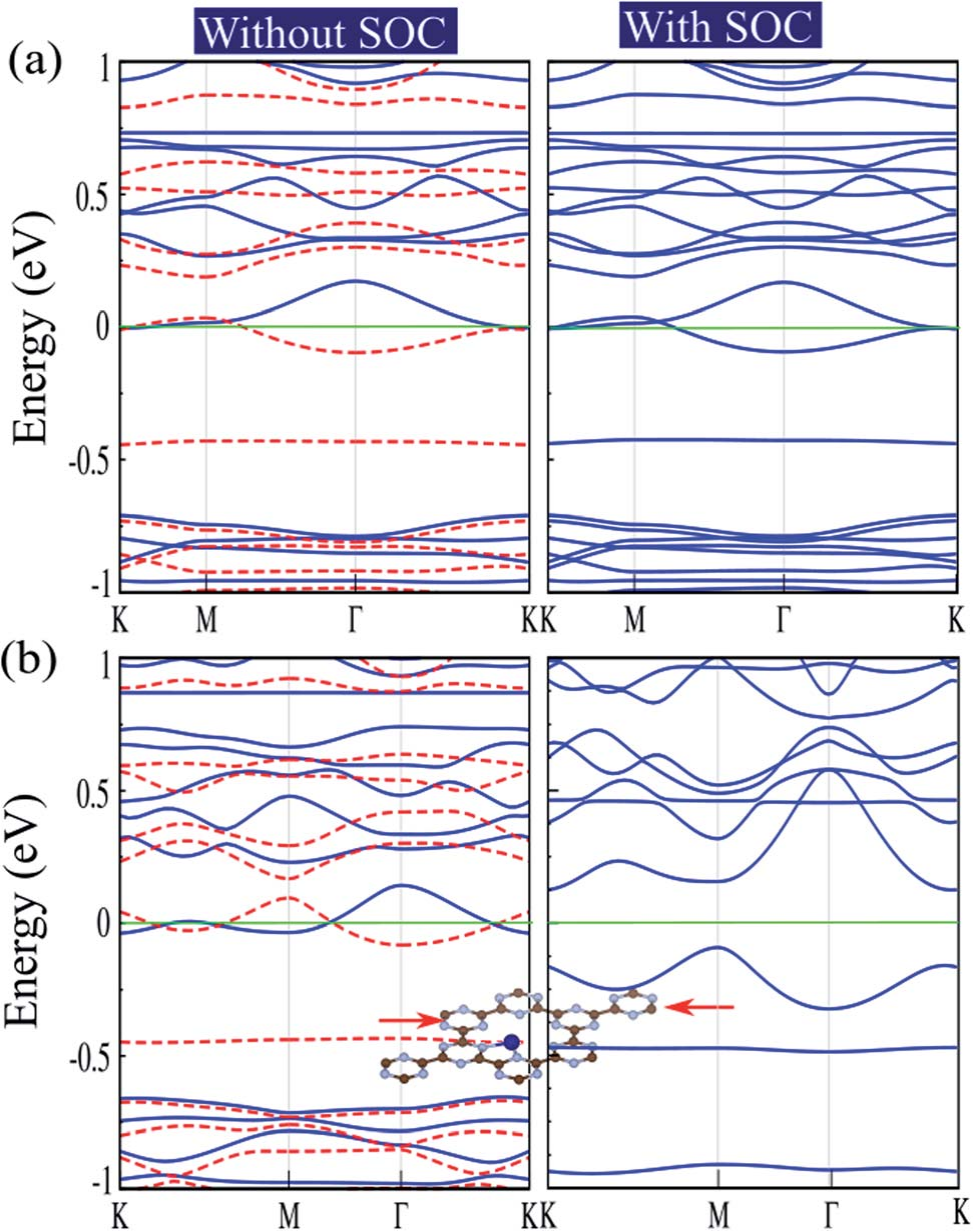
Electronic structures of (a) Cr@C_6_N_6_, without and with consideration of spin–orbital coupling (SOC) effect; (b) Cr@C_6_N_6_ upon −2% strain, without and with SOC effect. Optimized structures under the applied strain are shown in the insets. The energy bands of ↑ and ↓ spin channels are indicated by blue lines and red dashed lines, respectively. Zero energy is set at the Fermi-level.

The electronic structures of Cr@C_6_N_6_ without strain and with −2% strain, considering spin–orbital coupling (SOC) and without SOC, are shown in Fig. 7(a) and (b). For Cr@C_6_N_6_ upon −2% compression strain, without considering SOC, we can see that the ↑ and ↓ spin bands cross each other at the Fermi-level, which implies a magnetic semi-metallic nature. Our results show that when the SOC effect is considered, we see a touching point along the Γ and K paths at the Fermi level, and a narrow band gap of 125 meV forms in the proximity of the Fermi level, which implies a semi-metallic character. The effect of SOC correction on the evolution of electronic structure was found to be remarkable, such that degeneracies at the VBM are removed. Due to weak screening of the Coulomb interaction in the doping of transition metal atoms, the Hubbard U energy band structure may be expected to be weak. Since the accurate value of U has not been determined, we investigate the correlation effects by the value of the Hubbard U. To confirm that semi-metallicity is not an artificial result from the GGA+U method, we perform calculations for the doping of Cr atoms. The electronic band structures of Cr@C_6_N_6_ without strain and with −2% strain, without (left) and with consideration of the Hubbard U effect (right) are shown in Fig. S3(a) and (b),† respectively. The correlation effects on the electronic and magnetic properties of Cr@C_6_N_6_ without and with strain are significant and cause changes in the spin polarization. In the Cr-doped C_6_N_6_ without strain and upon −2% compression strain, we can see that the ↑ and ↓ spin bands cross each other at the Fermi-level, which implies a spin-polarized semi-metallic nature, without considering Hubbard U. We can see that a nodal-line forms around the K and M points when *U* = 2 eV is considered. Our results show that the magnetic moment of Cr@C_6_N_6_ in *U* = 2 eV are 3.53 *μ*_B_ (without strain) and 3.63 *μ*_B_ (with −2% strain).

Here, we discuss the magnetic coupling between Cr–Cr doped in C_6_N_6_ monolayers. We consider two Cr atoms doped in one and two neighboring cavities for comparison. The spin density differences of Cr doped in one and two neighboring cavities of C_6_N_6_ are shown in Fig. S4.† The electronic band structures are indicated in the right panels. All of the possible relative positions of two Cr atoms doped in one and two cavities of C_6_N_6_ are considered, and only the most stable configurations are exhibited. The exchange energy *E*_ex_, defined as *E*_ex_ = *E*_FM_ − *E*_AFM_, is calculated to determine the most stable phase. Negative and positive *E*_ex_ indicate the FM and AFM ground states, respectively. For the case of one neighboring cavity, it is found that the Cr atoms exhibit FM coupling (with *E*_ex_ = 140 meV) in their most stable phases, and the corresponding spin density differences are shown in Fig. S4(a).† Because the distance between the two Cr atoms is small (∼1.82 Å), one may think that the two Cr atoms should show direct coupling through C/N atoms. For the case of two cavities, it is clearly seen that the case of the Cr atoms is quite different from the above findings and they show AFM coupling (*E*_ex_ = 30 meV) with corresponding spin density differences.

## 5 Conclusion

In summary, using first-principles calculations, we have explored the effects of atom doping on the structural, electronic, and magnetic properties of C_6_N_6_ and C_6_N_8_ monolayers. Our theoretical results indicated that with atom doping, C_6_N_6_ becomes a semiconductor with a narrow band gap (Mg-doped), semi-metal (Ca-doped) or ferromagnetic metal (Cr-doped). Additionally, we applied mechanical strain to both monolayers with atoms doped in their lattices. Our first-principles results revealed that the band gap and magnetism can be finely tuned by strain as well. Surprisingly, when the compressive strain reaches 2%, Cr-doped C_6_N_6_ displays a phase transition to a spin-polarized semi-metal without SOC; when SOC is considered, a narrow band gap of 125 meV is opened. Notably, in the case of Cr atom doped C_6_N_6_ upon compressive strain, considering SOC, an unusual semi-metallic character can be observed. In the C-doped C_6_N_8_ between applied tensile strains of 0–8%, the band gap was found to increase monotonically from 70 meV (at 2%) to 200 meV (at 8%), while it exhibits semi-metallic behavior under compressive strain larger than 2%. Our results show that a magnetic-to-nonmagnetic phase transition can occur under large tensile strain in the Ca doped C_6_N_8_ monolayer. In addition, Mg and Ca atom doped C_6_N_6_ can show topological insulator states under strain. The results provided by our extensive theoretical analysis highlight very promising electronic properties of C_6_N_6_ and C_6_N_8_ monolayers doped with various atoms and under mechanical strain, and will hopefully serve as a guide for future experimental and theoretical studies.

## Conflicts of interest

The authors declare that there are no conflicts of interest regarding the publication of this paper.

## Supplementary Material

RA-010-D0RA04463F-s001
