# Supplementary material for: Investigation of strain and doping on the electronic properties of single layers of C6N6 and C6N8: a first principles study
Source: RSC Adv. 2020 Jul 24;10(46):27743–51. doi: 10.1039/d0ra04463f (PMC9055606; doi:10.1039/d0ra04463f)
Supplement: RA-010-D0RA04463F-s001 [file RA-010-D0RA04463F-s001.pdf]

Electronic Supplementary Material (ESI) for RSC Advances.
This journal is © The Royal Society of Chemistry 2020

Supplementary information// Investigation of doping and strain on the electronic properties of single layers of C6N6 and C6N8 : A first principles study

Asadollah Bafekry *[1,2], C. Nguyen[3] , Abbas Goudarzi [4], Mitra Ghergherehchi *[5] , Mohsen shafieirad[6]

[1] Department of Physics, University of Guilan, 41335-1914 Rasht, Iran

[2]Department of Physics, University of Antwerp, Groenenborgerlaan 171, B-2020 Antwerp, Belgium.

[3]Department of Materials Science and Engineering, Le Quy Don Technical University, Ha Noi 100000, Vietnam.

[4]Department of Physics University of North Texas Denton, Texas, USA.

[5]College of Electronic and Electrical Engineering, Sungkyun kwan University, Suwon, Korea;

[6]Department of Electrical and Computer Engineering, University of Kashan, Kashan, Iran

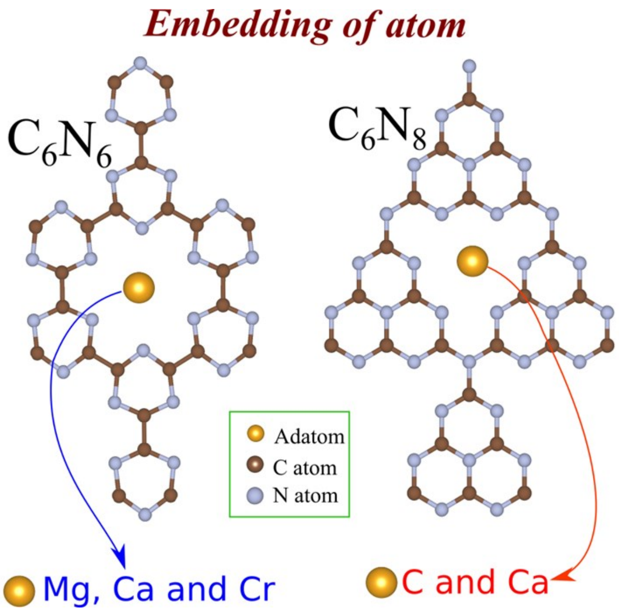

Fig. S1: : Schematic atomic structure for embedded atoms into C6N6 (left) and C6N8 (right).

A schematic view of the embedded atom into holey site of C6N6 and C6N8 monolayers are shown in Fig. S1. For the different atoms considered in our present study, the stable binding sites are uniformly at the nanopore.

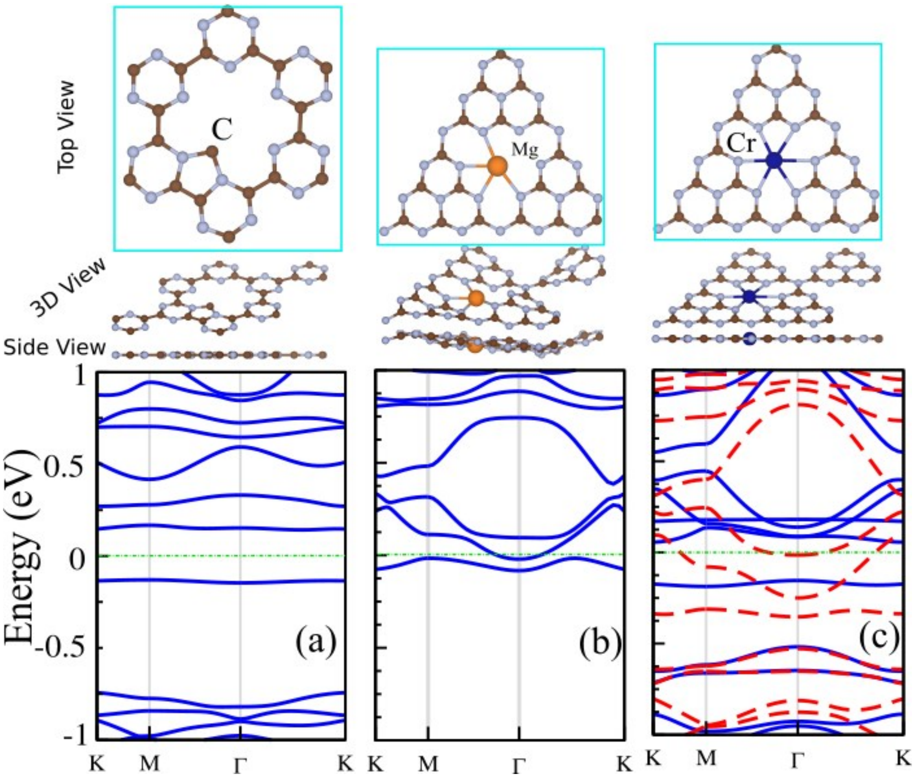

Fig. S2. Electronic structure of (a) C@C6N6 , (b) Mg@C6N8 and (e) Cr@C6N8. The zero of energy is set at Fermi-level. Top, 3D and side views of optimized structures of studied systems are shown in top of panel. The zero energy is set to the Fermi-level.

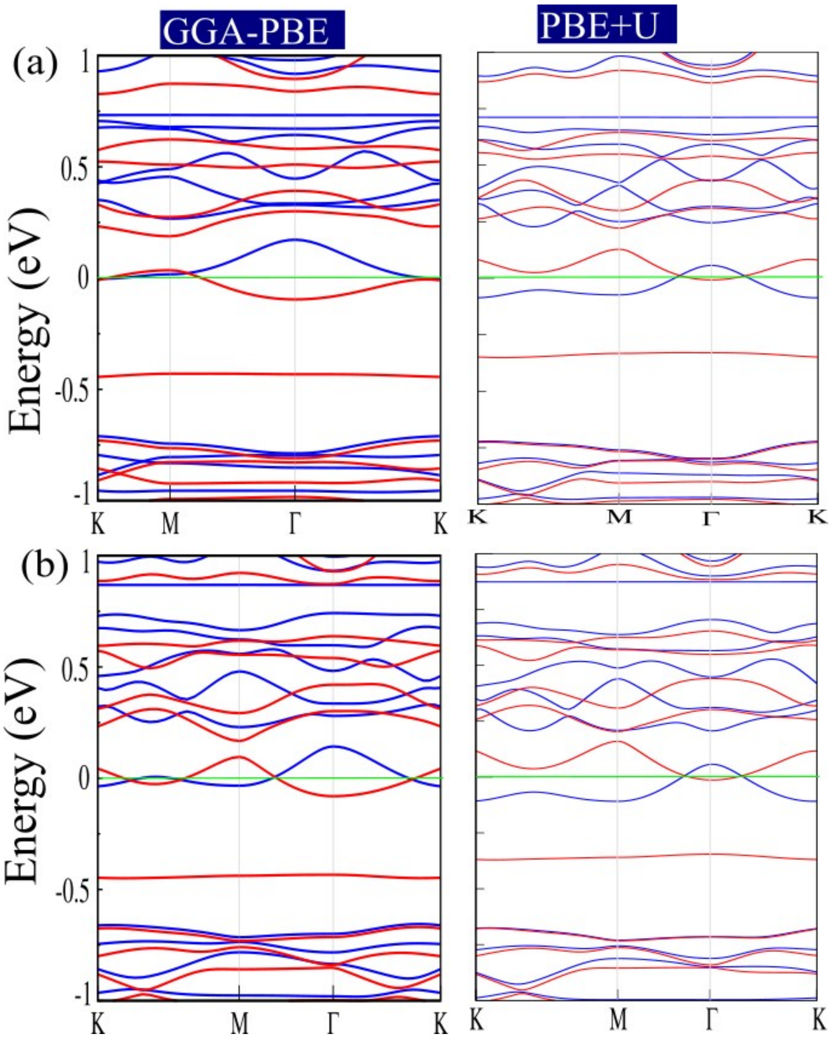

Fig. S3: Electronic structure of (a) Cr@ C6N6 , without and with considering of Hubbard U effect. (b) Cr@ C6N6 upon -2% strain, without and with U effect. The zero energy is set to the Fermi-level.

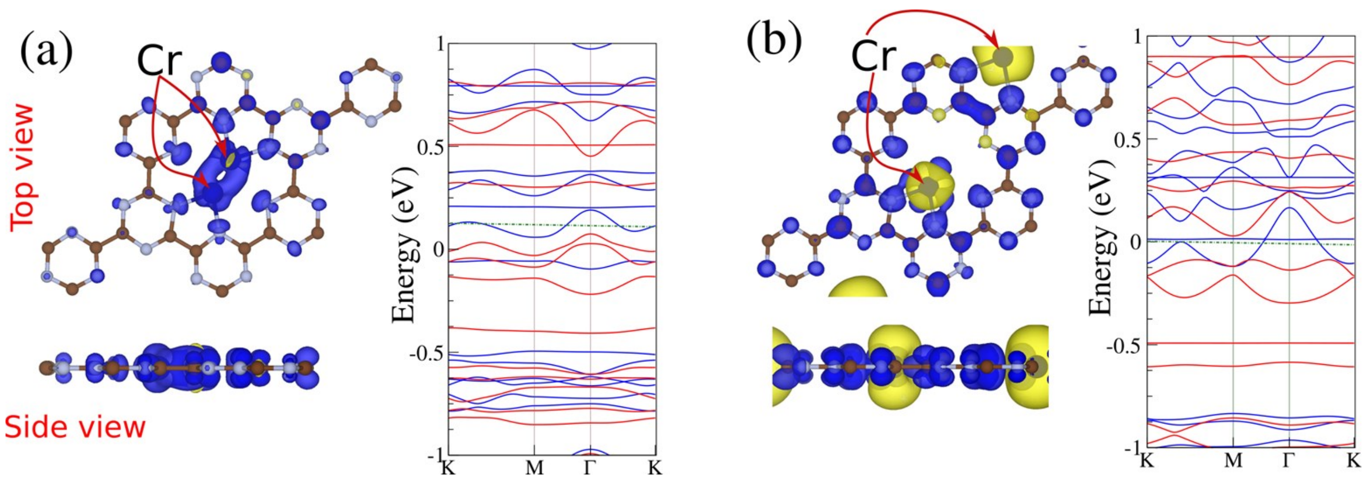

Fig. S4. Differences spin density of Cr embedded in (a) one and (b) two neighboring cavities of C_6N_6, positive and negative are represented by blue and yellow, respectively. Electronic band structure indicated in the left pannel.
